# Prevalence of Facebook Addiction in a Teenage Population: About 110 Cases

**DOI:** 10.1192/j.eurpsy.2022.2124

**Published:** 2022-09-01

**Authors:** N. Faouel, R. Ben Soussia, M. Gharbi, M. Kacem, A. Haj Mohamed, W. Bouali, L. Zarrouk

**Affiliations:** 1 hospital Tahar sfar Mahdia, Department Of Psychiatry Mahdia, Mahdia, Tunisia; 2 hospital Tahar sfar Mahdia, Epartment Of Psychiatry Mahdia, Mahdia, Tunisia; 3 hospital Tahar sfar Mahdia, Epartment Of Psychiatry Mahdia, monastir, Tunisia; 4 hospital Tahar sfar Mahdia, Department Of Psychiatry Mahdia, monastir, Tunisia; 5 University Hospital of Mahdia, Tunisia., Psychiatry, mahdia, Tunisia

**Keywords:** Tunisia, Addiction, teenagers, Facebook

## Abstract

**Introduction:**

Facebook use among Teenagers has become a very common phenomenon. Its use can resuly in Facebook addiction .

**Objectives:**

To estimate the prevalence of problematic Facebook use among a sample of school-going adolescents.

**Methods:**

This is a cross-sectional and descriptive study carried out among 110 school-going adolescenthe at 2 state colleges in Sidi Bouzid. We used a pre-established self-questionnaire containing 2 parts: a part exploring the socio-demographic data of the adolescent and a psychometric part: Bergen Facebook addiction Scale.

**Results:**

Study participants had a mean age of 14.4 years with extremes of 12 to 17 years. The sex ratio (M / F) (46/64) of the participants was 0.71.In our population, 13 students (11.8%) were smokers. Two students (1.8%) consumed alcohol. Cannabis use was noted in only one student.The majority of students (102), or 92.7%, had been online for more than a year.The daily Facebook connection time was more than 4 hours for 20.9%. Boredom was the number one reason for logging into Facebook for 82 students (74.5%) followed by curiosity for 45 students (40.9%). Fifteen students (13.6%) were addicted to Facebook (score> 10 on the Bergen Addiction Scale.

**Conclusions:**

Facebook can be a useful and interesting tool to maintain and develop a network of relationships and create new ones. Its problematic use or addiction to Facebook has become a new scourge of public health. Faced with the negative impact of this addiction, It would be necessary to rationalize this use.

**Disclosure:**

No significant relationships.

